# Vaccinating against the absent: Risks of the B/Yamagata strain in the live attenuated vaccine

**DOI:** 10.2807/1560-7917.ES.2024.29.49.2400766

**Published:** 2024-12-05

**Authors:** Parvaiz A Koul

**Affiliations:** 1 Pulmonary Medicine, Sher-i-Kashmir Institute of Medical Sciences, Soura, Srinagar, India

**Keywords:** Influenza, vaccine, Live vaccine, India, air-borne infections, viral infections, vaccines and immunisation

I read with interest the ‘perspective’ by Del Riccio et al., wherein the authors have discussed the implications of the World Health Organisation’s (WHO) recommendation of removing the influenza B/Yamagata virus from the seasonal influenza vaccine [1]. While the authors state that they agree in principle with the WHO stand on the transition from the quadrivalent to the trivalent forms of the vaccine based on reported surveillance, they caution that

“*seemingly overwhelming support provided by current epidemiological data to the hypothesis of B/Yamagata influenza virus disappearance must be counterbalanced by the consideration that the virus could still be circulating although at an extremely low level and therefore merely go undetected, while not yet gone extinct in the population. This could be the case if virus circulation were restricted to limited geographical regions in which surveillance is low or absent, especially considering the still low proportion of influenza B viruses for which the lineage is determined. In addition, it is not implausible that influenza B/Yamagata could re-emerge at some point in the future, just as influenza A(H1N1) re-emerged in 1978 two decades after it had been displaced by A(H2N2).*”

The authors further emphasise the strengthening of the surveillance systems and argue that, based on the lessons learnt from COVID-19,

“*reinforcing the global respiratory virus surveillance network would serve preparedness and alert purposes whose importance far exceeds that of providing the evidence necessary to declare the end of circulation of the B/Yamagata lineage influenza virus.”*

While these suggestions and arguments are indeed valid, it is prudent to reflect that the quadrivalent influenza vaccine was introduced subsequent to the trivalent vaccine at a time with an even lower level of surveillance, and that continuing vaccination against the B/Yamagata virus is tantamount to vaccination against an absent virus. This way, we will need to vaccinate against all viruses regardless of their documented circulation. I am not sure that many researchers would subscribe to that view. I certainly do not.

The global circulation of the B/Yamagata lineage of the influenza virus has diminished drastically since 2020, prompting questions regarding its continued inclusion in future influenza vaccines [2,3]. In its latest recommendations for the 2025 influenza vaccine composition for southern hemisphere, the WHO advised against the inclusion of the B/Yamagata virus, against the backdrop of all circulating detections of influenza B being of the B/Victoria lineage. Thus, the inclusion of the B/Yamagata antigen in the influenza vaccine is no longer justified [3]. However, the WHO also advised that national or regional authorities should determine whether to transition to trivalent influenza vaccines based on local circumstances. In case a decision is made to use quadrivalent vaccines, the B/Yamagata lineage component would remain unchanged from previous recommendations: a B/Phuket/3073/2013 (B/Yamagata lineage)-like virus [4].

Apart from the argument that B/Yamagata does not circulate and hence should not be included in the vaccine, concerns have been raised about re-introduction of the B/Yamgata virus into the community [2,3]. These concerns were reinforced following a recent report of a young child with lymphoblastic leukaemia who had developed a B/Yamagata influenza infection. Genetic analysis confirmed that the infectious agent was identical to the quadrivalent live attenuated influenza vaccine (LAIV) the child had received before the leukaemia diagnosis [5]. Such scenarios are distinctly possible with the administration of live vaccines to any recipient who could have an undiagnosed immunosuppressed status. Although such reintroductions could also occur during the manufacturing process of inactivated vaccines, the risk is notably higher with the administration of LAIV.

Notably, the Advisory Committee on Immunization Practices (ACIP) in the United States has recommended that all influenza vaccines available during the 2024/25 season be trivalent [6]. Similarly, the European Union has emphasised that live influenza vaccines for the 2024/25 season should be trivalent and exclude the B/Yamagata strain [7]. However, quadrivalent LAIV continues to be used in some countries including India, Canada etc, presenting a clear risk of reintroducing the strain into community circulation. This situation is reminiscent of vaccine-derived poliovirus infections. In the context of low influenza vaccine uptake in low- and middle-income countries, there is a potential for mutations in a B/Yamagata-naïve population, which could contribute to the reintroduction of the mutated strain into the community.

Given these circumstances, I think that there is an urgent need to recall LAIV containing the B/Yamagata virus, to eliminate the risks of vaccine-induced infections and reintroduction of the virus into the community. If trivalent LAIV is unavailable, vaccinators would rather opt for an inactivated vaccine than for the LAIV.
